# The Effect of Sodium–Glucose Cotransporter-2 Inhibitors on COVID-19 Patients with Type 2 Diabetes Mellitus: A Retrospective Cohort Study Using the Common Data Model

**DOI:** 10.3390/jcm13020431

**Published:** 2024-01-12

**Authors:** Kyoung Ree Lim, Kwang Jin Chun, Bum Sung Kim, Seunghwa Lee

**Affiliations:** 1 Division of Infectious Diseases, Department of Internal Medicine, Kyung Hee University Hospital at Gangdong, Seoul 05278, Republic of Korea; idkrlim@naver.com; 2 Division of Cardiology, Department of Internal Medicine, Kangwon National University Hospital, Kangwon National University School of Medicine, Chuncheon 24341, Republic of Korea; imchunn@naver.com; 3 Division of Cardiology, Department of Medicine, Konkuk University Medical Center, Seoul 05029, Republic of Korea; dolphindance98@gmail.com; 4 Division of Cardiology, Department of Medicine, Wiltse Memorial Hospital, Suwon 16480, Republic of Korea

**Keywords:** SGLT2 inhibitor, COVID-19, MACCE

## Abstract

Background: There is no clinical evidence about the effect of sodium–glucose cotransporter-2 (SGLT2) inhibitors on diabetic patients who have been diagnosed with coronavirus disease 19 (COVID-19). Methods: The dataset is based on insurance benefit claims sent to the Health Insurance Review and Assessment Service of Korea from January, 2018 to April, 2022. Among 9,822,577 patients who were involved in the claims, diabetic patients were divided into two groups based on whether they had a prescription for an SGLT2 inhibitor. The primary outcome was major adverse cardiac and cerebrovascular events (MACCEs), which were a composite of all-cause mortality, myocardial infarction, stroke, and revascularization over 90 days. Results: A total of 172,682 patients was analyzed. In the propensity score-matched analysis, the rate of MACCE was lower in the SGLT2 inhibitor group compared to the non-SGLT2 inhibitor group (0.89% vs. 1.31%; hazard ratio, 0.71; 95% confidence interval, 0.53–0.94; *p* =0.020). Each of the MACCEs showed no differences between the two groups. The rate of pneumonia was similar between the two groups (4.45% vs. 4.39%; hazard ratio, 1.06; 95% confidence interval, 0.91–1.16; *p* = 0.620). Conclusions: In the diabetic patients who were diagnosed with COVID-19, SGLT2 inhibitors were associated with improved clinical outcomes in terms of MACCEs. SGLT2 inhibitors might be considered for prescription to diabetic patients in the current context of long COVID-19.

## 1. Introduction

The COVID-19 pandemic has brought significant challenges to healthcare systems worldwide. Despite global efforts to move past the acute phase of the pandemic, it is estimated that at least 65 million people suffer from long COVID-19—a post-infection condition with several symptoms that can inhibit daily activities for months or even years [1]. Among those most affected are individuals with pre-existing conditions such as diabetes or coronary artery disease [2,3]. Moreover, diabetic patients seem to be at greater risk of severe COVID-19 infection, compared to other chronic conditions [4]. COVID-19 exposure is associated with an increased risk of adverse cardiovascular events, especially in patients requiring hospitalization and in the early post-infection period [5]. Moreover, worse cardiovascular outcomes were not confined only within the acute illness phase, but were shown even after 12 months [6].

Sodium–glucose co-transporter 2 (SGLT2) inhibitors are a class of drugs that lower blood sugar levels by preventing the kidneys from reabsorbing glucose. They have been proven effective in the treatment of type 2 diabetes mellitus (DM), various forms of heart failure, and kidney failure, and represent one of the major pharmacological advances in cardiovascular medicine in the 21st century [7]. These drugs have been shown to reduce the risk of cardiovascular events in diabetic patients [8]. Several studies on the association between SGLT2 inhibitors and COVID-19 exist [9,10,11]. Although SGLT2 inhibitors could be safely prescribed to diabetic patients with COVID-19, randomized trials have shown that treatment with an SGLT2 inhibitor was not associated with improved clinical outcomes [11]. However, there is a limitation due to the low event rate and small study population; moreover, the population studied were patients with cardiometabolic risk factors, rather than diabetic patients.

Therefore, we aimed to evaluate whether the use of SGLT2 inhibitors affects improved cardiovascular outcomes in diabetic patients with COVID-19 using de-identified COVID-19 nationwide data from the Republic of Korea. Our findings may provide additional insight into the prescription of SGLT2 inhibitors.

## 2. Materials and Methods

The current dataset, based on insurance benefit claims sent to the Health Insurance Review and Assessment Service of Korea (HIRA), is composed of all the patients who used National Health Insurance of Korea from January 2018 to April 2022. Among those people, 9,822,577 patients were selected and converted to the Observational Medical Outcomes Partnership (OMOP)–common data model (CDM) by the Big Data Department of HIRA. The process of standardizing OMOP-CDM from insurance benefit claims is described elsewhere [12]. The name of the database is HIRA_CMD, and the used platform is Oracle. We used the database shared in the form of OMOP-CDM, which has been established as a multi-stakeholder, interdisciplinary collaborative to create open-source solutions that bring out the value of observational health data through large-scale analytics [13].

### 2.1. Definitions and Outcomes

Diabetic patients who were diagnosed with COVID-19 were divided into two cohorts. The target cohort consisted of patients with an SGLT2 inhibitor prescription claim within 6 months before their COVID-19 diagnosis, and the comparative cohort consisted of all other patients without an SGLT2 inhibitor prescription. To avoid duplication of patients, the patients with the first infection were enrolled, and reinfections were excluded. Baseline characteristics were retrieved from OMOP-CDM of HIRA. The primary outcome was major adverse cardiac and cerebrovascular events (MACCEs), defined as a composite of all-cause death, myocardial infarction, stroke, or revascularization within 90 days of COVID-19 diagnosis. Secondary outcomes were each of the composite elements and occurrence of heart failure within 90 days of COVID-19 diagnosis. Additionally, to evaluate the effect of SGLT2 inhibitors on infection, the occurrence rates of pneumonia and sepsis within 90 days after COVID-19 diagnosis were also analyzed. We analyzed clinical events within 90 days because a previous meta-analysis about COVID-19 patients showed that most symptoms suggestive of a cardiac event, such as chest pain or dyspnea, occurred within 90 days [14].

### 2.2. Statistical Analysis

Analysis tools of OMOP-CDM are built in the interactive analysis platform ATLAS and the Observational Health Data Sciences and Informatics (OHDSI) Methods Library R packages. OHDSI’s open-source software is publicly available on the GitHub repository (https://github.com/OHDSI/ accessed on 20 December 2022). In addition, concept sets which we used to define baseline characteristics and study outcomes are also available (https://github.com/OHDSI/COVID-19/ accessed on 20 December 2022). We performed logistic regression to analyze MACCEs and other clinical outcomes. Kaplan–Meier estimates were used to construct survival curves, and compared with the log-rank test. Cox regression was used to evaluate MACCEs associated with the use of an SGLT2 inhibitor. To retain a large sample size and maximize the study power while maintaining a balance in covariates between the two groups, we conducted rigorous adjustment for differences in baseline and lesion characteristics of patients using the weighted Cox proportional hazard regression model with propensity score (PS) stratification and PS matching with caliper 0.2, and generated a population to match the cohorts without sample replacement [15]. Variables retained in the matching included age; female sex; and diagnosis of infectious, gastrointestinal, respiratory, endocrinal, cardiac or malignant disease codes with non-zero coefficients during 1 year prior to the diagnosis of COVID-19. The propensity score was stratified into five strata, and the Cox regression analysis retained strata. All tests were two-tailed, and *p* < 0.05 was considered statistically significant.

## 3. Results

### 3.1. Cohort Characteristics

**Figure 1:**
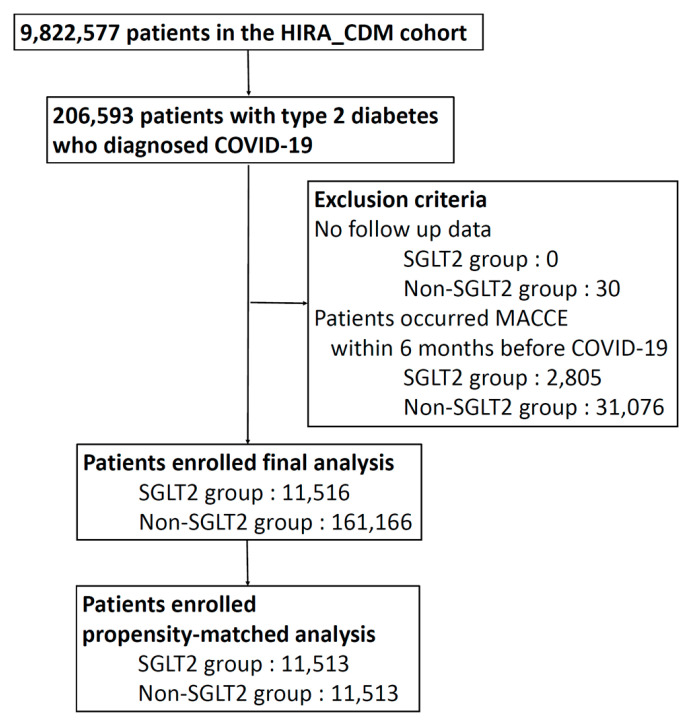
Attrition of analysis of major cardiac and cardiovascular events.

A total of 172,682 diabetic patients who were diagnosed with COVID-19 were included in the analysis of MACCEs, among which 11,516 patients were prescribed SGLT2 inhibitors during the last six months before COVID-19 diagnosis, and 161,166 patients were not prescribed SGLT inhibitor (Figure 1). We created 11,513 matched pairs of patients via propensity score-matching for the entire cohort. Table 1 shows the baseline characteristics in the analysis of MACCEs. Within the entire cohort, the SGLT-2 inhibitor group showed a higher incidence of hyperlipidemia, hypertensive disorder, heart failure, ischemic or coronary heart disease, and peripheral vascular disease. We found no significant differences in the baseline variables of the PS-matched population between groups (Supplementary Figure S1); in terms of pneumonia diagnosis, a total of 12,400 matched pairs were generated, and showed no difference after propensity matching (Table 2, Supplementary Figures S2 and S3).

### 3.2. Clinical Outcomes

**Figure 2:**
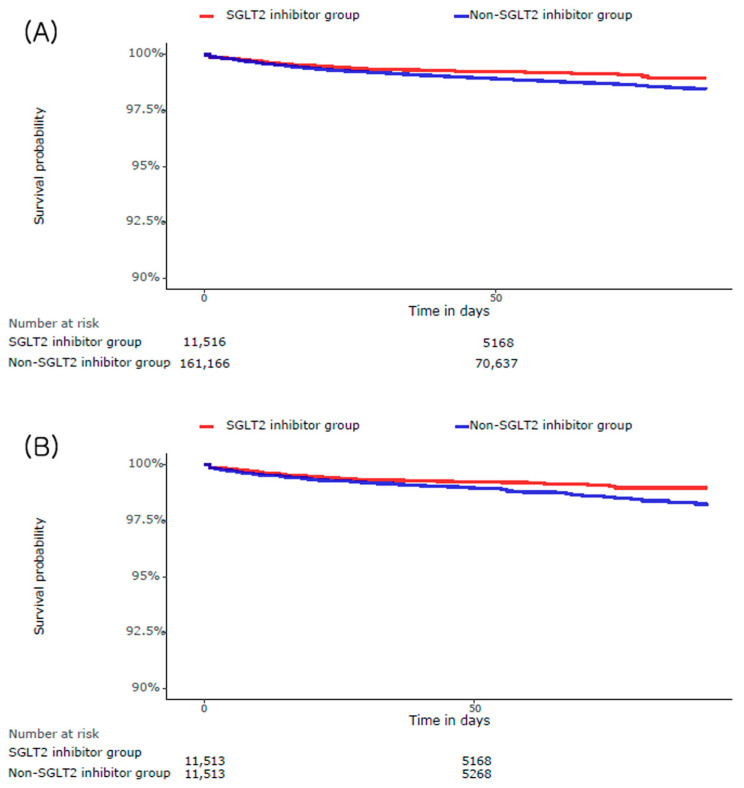
Kaplan–Meier curves for MACCEs in the (**A**) crude population and (**B**) propensity score-matched population.

The median follow-up duration for MACCEs was 48 days (interquartile range (IQR 39–73)) in the SGLT2 inhibitor group and 47 days (IQR, 39–72) in the non-SGLT2 inhibitor group. During the crude analysis, the incidence of MACCEs was lower in the SGLT2 inhibitor than that in the non-SGLT2 inhibitor group [0.89% vs. 1.31%; hazard ratio (HR), 0.70; confidence interval (CI) 95%, 0.57–0.85; *p* < 0.01] (Table 3, Figure 2). Analysis of the PS-matched cohort showed similar results for MACCEs (0.89% vs. 1.31%; HR, 0.71; CI 95%, 0.53–0.94; *p* = 0.02). The rate of all-cause death was also lower in the SGLT2 inhibitor group (1.10% vs. 1.51%; HR, 0.72; CI 95%, 0.61–0.81; *p* < 0.01), but did not differ significantly during the PS-matched analysis (1.10% vs. 1.35%; HR, 0.80; CI 95%, 0.64–1.01; *p* = 0.06) (Table 3).

Regarding pneumonia occurrence, there was no significant difference between the SGLT2 and non-SGLT2 inhibitor groups in either the crude (4.5% vs. 4.2%; HR, 1.06; CI 95%, 0.97–1.15; *p* = 0.20) or PS-matched analysis (4.5% vs. 4.4%; HR, 1.03; CI 95%, 0.91–1.16; *p* = 0.62) (Table 4, Supplementary Figure S4). The incidence of sepsis was similar between the two groups in both the crude (0.23% vs. 0.31%; HR, 0.76; CI 95%, 0.53–1.07; *p* = 0.13) and PS-matched analysis (0.23% vs. 0.28%; HR, 0.78; CI 95%, 0.46–1.31; *p* = 0.36) (Table 4).

## 4. Discussion

The results of the present study are as follows. First, SGLT2 inhibitor treatment showed improved short-term cardiovascular events in diabetic patients diagnosed with COVID-19 compared to patients without SGLT2 inhibitor. Second, SGLT2 inhibitor treatment did not reduce the progress of infections such as pneumonia or sepsis. Considering the results of the present study, the beneficial effect of SGLT2 inhibitors on the general population was found to be similar in COVID-19 patients with diabetes.

Diabetic patients with COVID-19 are disproportionately affected, with an increased risk of hospitalization and mortality [16]. Several additional concerns exist, such as appropriate glucose-lowering therapy, new-onset diabetes, and increased diabetic ketoacidosis [17,18]. As the coronavirus infection moves from pandemic to endemic, treatment strategies for long COVID-19 are also required for these patients [1].

We already know that SGLT2 inhibitors positively impact various pathways that are disrupted during acute illness, such as inhibiting glycolysis and stimulating lipolysis, and reducing oxidative stress and inflammation [11,19]. In this regard, the DARE-19 trial was conducted, and showed that dapagliflozin treatment showed no benefit in reducing the risk of organ dysfunction or death, or improvement in clinical recovery in patients with cardiometabolic risk [11]. Meanwhile, SGLT2 inhibitors have shown benefits in reducing cardiovascular outcomes in patients, including those with heart failure, chronic kidney disease, and coronary artery disease [7,8,20]. Given that COVID-19 patients have an increased incidence of cardiovascular disease and cardiovascular death, these patients may benefit from reduced cardiovascular events, but not from infectious events [3,5,6,21]. However, there are limited data on the association between SGLT2 inhibitors and cardiovascular outcomes in diabetic patients diagnosed with COVID-19. This paper is notable because there is no clinical evidence about the effect of SGLT2 inhibitors on diabetic patients who have been diagnosed with COVID-19.

The results of the current study showed that diabetic patients with SGLT2 inhibitor treatment had improved cardiovascular outcomes compared to patients without SGLT2 inhibitor treatment. This result correlates well with current evidence [3,5,6,21]. COVID-19 patients with increased cardiovascular risk have increased mortality. Although the mechanism of cardiac injury in COVID-19 is not well known, several studies suggested that extreme stress secondary to acute pulmonary disease or inflammation-related thrombosis due to viral infection may contribute to cardiac injury and worse cardiovascular outcomes [22,23]. The suggested mechanisms by which SGLT2 inhibitors benefit the cardiovascular system include a reduction in adipose tissue-mediated inflammation and pro-inflammatory cytokine production, a shift towards ketone bodies as the metabolic substrate for the heart, reduced oxidative stress, lowered serum uric acid levels, and suppression of advanced glycation end-product signaling [24]. Intriguingly, SGLT2 inhibitors improved relatively short-term clinical outcomes within 90 days in the present study. In addition, the incidence of HF was also reduced in the SGLT2 inhibitor group. In light of the results of this study, SGLT2 inhibitors might be prescribed to DM patients in the current endemic COVID situation.

Analysis of infection events such as pneumonia or sepsis showed that SGLT2 inhibitor treatment did not provide benefits. Due to the inherent effects of SGLT2 inhibitors such as anti-inflammatory effects and cellular protection, there was an expectation that SGLT2 inhibitors may improve the clinical course of COVID-19 patients. A recent study showed that the risk of sepsis and pneumonia was lower in the diabetic patients who began with an SGLT2 inhibitor, compared to dipeptidyl peptidase 4 inhibitors [25]. Despite this, risk reduction due to SGLT2 inhibitors was not found in the COVID-19 patients [11,26]. In particular, most pneumonia cases occurred at the time of COVID-19 diagnosis in the present study. Hence, it could be assumed that SGLT2 inhibitors have no preventive effect on acute infection. The results of the current study provide further evidence that SGLT2 inhibitors might have no beneficial effect on additional bacterial infections such as pneumonia or sepsis in COVID-19 patients.

The results of this study should be interpreted considering the following limitations. First, this is a retrospective study. Despite our efforts to adjust all confounding factors by PS stratification and matching analysis, unmeasured factors might have affected the results. Second, owing to the nature of the database that was retrieved from insurance-issued claims, clinical presentation, symptoms, and hospital course could not be evaluated. In addition, we could not analyze the effect according to SARS-CoV-2 subtype, because CDM does not provide information about the time of infection. Third, the continuation or discontinuation of the SGLT2 inhibitor during the infection period could not be analyzed. Moreover, other diabetes drugs such as metformin or insulin were not included in the analysis. In addition, the CDM dose not provide each type of SGLT2 inhibitor; thus, the effect of each SGLT2 inhibitor could not be evaluated. The results of the current study were derived from a cohort in the Republic of Korea, hence the impact of ethnicity cannot be analyzed, and further evaluation is needed. Finally, occurrence of urinary tract infection—one of the concerns when using SGLT2 inhibitors—was not evaluated. Despite these limitations, this study provides real-world evidence about the use of SGLT2 inhibitors and clinical outcomes in DM patients diagnosed with COVID-19, as well as valuable information in the current long COVID situation.

## 5. Conclusions

In diabetic patients who were diagnosed with COVID-19, SGLT2 inhibitor treatment showed benefits for cardiovascular outcomes, but not for pneumonia or sepsis. SGLT2 inhibitor treatment might be considered for diabetic patients in the current long COVID-19 situation.

## Figures and Tables

**Table 1:** Baseline characteristics of the analysis for MACCEs.

	Before PS Adjustment			After PS Adjustment		
	SGLT2 Inhibitor	Non-SGLT2 Inhibitor	SMD	SGLT2 Inhibitor	Non-SGLT2 Inhibitor	SMD
	(*n* = 11,516)	(*n* = 161,166)		(*n* = 11,513)	(*n* = 11,513)	
Age group						
15–19	0.1	0.6	−0.09	0.1	0.2	−0.03
20–24	0.6	0.9	−0.04	0.7	0.6	0
25–29	0.9	1.4	−0.04	1	0.9	0.01
30–34	1.7	2	−0.03	1.7	1.5	0.02
35–39	3.6	3.3	0.02	3.7	3.6	0.01
45–49	8.2	6	0.09	8.4	8.1	0.01
50–54	11	8.6	0.08	11.3	11	0.01
60–64	17	15.1	0.05	17	17	0
65–69	14	14.3	−0.01	13.9	13.7	0.01
70–74	10.4	11.3	−0.03	10.2	10.2	0
75–79	6.4	8.2	−0.07	6.1	6.5	−0.01
80–84	3.9	6.7	−0.13	3.5	3.7	−0.01
85–89	1.6	3.9	−0.14	1.4	1.5	−0.01
90–94	0.4	1.5	−0.1	0.4	0.5	−0.02
95–99	0.1	0.3	−0.06	0.1	0.2	−0.04
Female sex	47.7	54.1	−0.13	47.9	46.8	0.02
Medical history						
Acute respiratory disease	79.8	79.4	0.01	80.2	79.5	0.02
Chronic liver disease	8.2	7.4	0.03	8.1	8.3	−0.01
Chronic obstructive lung disease	2.6	2.7	−0.01	2.3	2.6	−0.02
Crohn’s disease	0.1	0.1	−0.01	0.1	0.1	0
Dementia	5.5	9.3	−0.14	4.8	5.1	−0.01
Depressive disorder	12.7	14.5	−0.05	12.1	11.7	0.01
Gastroesophageal reflux disease	41.4	40.8	0.01	40.8	40.6	0
Gastrointestinal hemorrhage	3	3.3	−0.01	2.8	2.8	0
Hyperlipidemia	83.8	68.3	0.37	83.7	84.9	−0.03
Hypertensive disorder	66.9	56.8	0.21	65.9	67.2	−0.03
Lesion of liver	5.1	4.7	0.02	5	5.3	−0.01
Obesity	0.7	0.3	0.06	0.8	0.6	0.02
Osteoarthritis	23.3	24.1	−0.02	23.1	22.6	0.01
Pneumonia	5.6	5.7	−0.01	5	4.9	0
Psoriasis	1.4	1	0.04	1.4	1.1	0.02
Schizophrenia	1	1.4	−0.03	1	1.2	−0.02
Urinary tract infectious disease	7.2	7.1	0	6.6	6.3	0.01
Visual system disorder	51.6	47.8	0.08	51.4	50.9	0.01
Cardiovascular disease						
Atrial fibrillation	3.5	2.2	0.07	3.1	3	0.01
Cerebrovascular disease	8.9	7.8	0.04	8.4	8.5	0
Coronary arteriosclerosis	4.8	2.2	0.14	4.6	4.4	0.01
Heart disease	30.7	22.2	0.19	29.2	30.1	−0.02
Heart failure	12.7	8.3	0.14	11.5	11.6	0
Ischemic heart disease	20.1	12.8	0.2	19.2	20.1	−0.02
Peripheral vascular disease	25.5	18.8	0.16	24.9	25.3	−0.01
Pulmonary embolism	0.7	0.7	0	0.5	0.6	−0.01
Venous thrombosis	1.5	1.5	0	1.4	1.3	0.01
Neoplasms						
Hematologic neoplasm	0.5	0.6	−0.01	0.5	0.7	−0.02
Malignant lymphoma	0.1	0.2	−0.02	0.1	0.2	−0.02
Malignant neoplasm of anorectum	0.3	0.3	−0.01	0.3	0.4	−0.03
Malignant neoplastic disease	8.1	8.4	−0.01	7.9	8.3	−0.01
Malignant tumor of breast	0.8	0.8	0	0.8	0.7	0.01
Malignant tumor of colon	0.6	0.7	0	0.6	0.7	−0.01
Malignant tumor of lung	0.3	0.4	−0.01	0.3	0.4	−0.03
Malignant tumor of urinary bladder	0.2	0.3	−0.02	0.2	0.3	−0.02
Malignant neoplasm of prostate	0.9	1	−0.01	0.8	0.9	0

Data are presented as %. PS, propensity score; SGLT2, sodium glucose cotransporter 2; SMD, standardized mean difference.

**Table 2:** Baseline characteristics of the analysis for pneumonia.

	Before PS Adjustment			After PS Adjustment		
	SGLT2 Inhibitor	Non-SGLT2 Inhibitor	SMD	SGLT2 Inhibitor	Non-SGLT2 Inhibitor	SMD
	(*n* = 12,401)	(*n* = 164,471)		(*n* = 12,400)	(*n* = 11,513)	
Age group						
15–19	0.1	0.6	−0.09	0.1	0.2	−0.03
20–24	0.6	0.9	−0.04	0.7	0.7	0.01
25–29	0.9	1.4	−0.04	1	0.8	0.02
30–34	1.7	2	−0.03	1.7	1.4	0.03
35–39	3.6	3.3	0.02	3.6	3.3	0.01
40–44	6.8	5.4	0.06	7.1	7	0
45–49	8.2	6	0.09	8.6	8.6	0
50–54	11	8.6	0.08	11.6	11.6	0
55–59	13.3	10.1	0.1	13.9	14	0
60–64	17	15.1	0.05	17.2	16.9	0.01
65–69	14	14.3	−0.01	13.8	13.7	0
70–74	10.4	11.3	−0.03	9.8	9.9	0
75–79	6.4	8.2	−0.07	5.8	6.1	−0.01
80–84	3.9	6.7	−0.13	3.4	3.5	−0.01
85–89	1.6	3.9	−0.14	1.3	1.5	−0.02
90–94	0.4	1.5	−0.1	0.3	0.4	−0.02
Female sex	47.7	54.1	−0.13	46.8	46.2	0.01
Medical history: General						
Acute respiratory disease	79.8	79.4	0.01	79.7	78.8	0.02
Chronic liver disease	8.2	7.4	0.03	8	8.4	−0.02
Chronic obstructive lung disease	2.6	2.7	−0.01	1.8	2.2	−0.03
Crohn’s disease	0.1	0.1	−0.01	0.1	0.1	−0.01
Dementia	5.5	9.3	−0.14	4.3	4.6	−0.01
Depressive disorder	12.7	14.5	−0.05	11.2	10.9	0.01
Gastroesophageal reflux disease	41.4	40.8	0.01	39.7	39.5	0
Gastrointestinal hemorrhage	3	3.3	−0.01	2.7	2.5	0.01
Hyperlipidemia	83.8	68.3	0.37	83.8	85.6	−0.05
Hypertensive disorder	66.9	56.8	0.21	65.8	67.7	−0.04
Lesion of liver	5.1	4.7	0.02	5.1	5.3	−0.01
Obesity	0.7	0.3	0.06	0.7	0.6	0.02
Osteoarthritis	23.3	24.1	−0.02	22.5	22.2	0.01
Pneumonia	5.6	5.7	−0.01	1.9	1.6	0.02
Psoriasis	1.4	1	0.04	1.4	1.2	0.02
Renal impairment	6.5	5.4	0.04	5.6	5.7	−0.01
Rheumatoid arthritis	2.4	2.9	−0.03	2.2	2.2	0
Schizophrenia	1	1.4	−0.03	0.9	1.2	−0.03
Ulcerative colitis	0.1	0.2	−0.01	0.1	0.1	−0.01
Urinary tract infectious disease	7.2	7.1	0	6.5	6	0.02
Visual system disorder	51.6	47.8	0.08	50.9	50.4	0.01
Medical history: Cardiovascular disease						−0.02
Atrial fibrillation	3.5	2.2	0.07	3	3	0
Cerebrovascular disease	8.9	7.8	0.04	8.2	8.3	0
Coronary arteriosclerosis	4.8	2.2	0.14	4.6	4.4	0.01
Heart disease	30.7	22.2	0.19	28.8	29.9	−0.02
Heart failure	12.7	8.3	0.14	11.2	11.4	−0.01
Ischemic heart disease	20.1	12.8	0.2	18.8	19.8	−0.02
Peripheral vascular disease	25.5	18.8	0.16	24.6	24.9	−0.01
Pulmonary embolism	0.7	0.7	0	0.5	0.5	0
Venous thrombosis	1.5	1.5	0	1.4	1.3	0.01
Medical history: Neoplasms						
Hematologic neoplasm	0.5	0.6	−0.01	0.5	0.7	−0.03
Malignant lymphoma	0.1	0.2	−0.02	0.1	0.1	−0.01
Malignant neoplasm of anorectum	0.3	0.3	−0.01	0.3	0.5	−0.03
Malignant neoplastic disease	8.1	8.4	−0.01	7.7	8.1	−0.02
Malignant tumor of breast	0.8	0.8	0	0.8	0.8	0
Malignant tumor of colon	0.6	0.7	0	0.6	0.7	−0.01
Malignant tumor of lung	0.3	0.4	−0.01	0.2	0.3	−0.02
Malignant tumor of urinary bladder	0.2	0.3	−0.02	0.2	0.3	−0.01
Malignant neoplasm of prostate	0.9	1	−0.01	0.9	0.8	0

Data are presented as %. PS, propensity score; SGLT2, sodium glucose cotransporter 2; SMD, standardized mean difference.

**Table 3:** Clinical outcomes for MACCEs and other secondary outcomes.

	Crude Population				Propensity Score-Matched Population			
MACCE	SGLT2 inhibitor (*n* = 11,516)	Non-SGLT2 inhibitor (*n* = 161,166)	Unadjusted HR (95% CI)	*p* value	SGLT2 inhibitor (*n* = 11,513)	Non-SGLT2 inhibitor (*n* = 11,513)	Adjusted HR (95% CI)	*p* value
	103 (0.89)	2057 (1.28)	0.70 (0.57–0.85)	<0.01	103 (0.89)	151 (1.31)	0.71 (0.53–0.94)	0.02
All-cause death	SGLT2 inhibitor (*n* = 14,319)	Non-SGLT2 inhibitor (*n* = 192,216)	Unadjusted HR (95% CI)	*p* value	SGLT2 inhibitor (*n* = 14,313)	Non-SGLT2 inhibitor (*n* = 14,313)	Adjusted HR (95% CI)	*p* value
	157 (1.10)	2901 (1.51)	0.72 (0.61–0.84)	<0.01	157 (1.10)	193 (1.35)	0.80 (0.64–1.01)	0.06
Myocardial infarction	SGLT2 inhibitor (*n* = 13,591)	Non-SGLT2 inhibitor (*n* = 187,149)	Unadjusted HR (95% CI)	*p* value	SGLT2 inhibitor (*n* = 13,589)	Non-SGLT2 inhibitor (*n* = 13,589)	Adjusted HR (95% CI)	*p* value
	16 (0.12)	223 (0.12)	0.98 (0.57–1.58)	0.95	16 (0.12)	26 (0.19)	0.68 (0.35–1.30)	0.26
Stroke	SGLT2 inhibitor (*n* = 12,435)	Non-SGLT2 inhibitor (*n* = 167,593)	Unadjusted HR (95% CI)	*p* value	SGLT2 inhibitor (*n* = 12,426)	Non-SGLT2 inhibitor (*n* = 12,426)	Adjusted HR (95% CI)	*p* value
	23 (0.18)	395 (0.24)	0.78 (0.50–1.16)	0.25	23 (0.18)	44 (0.35)	0.62 (0.32–1.18)	0.16
Heart failure	SGLT2 inhibitor (*n* = 11,671)	Non-SGLT2 inhibitor (*n* = 166,606)	Unadjusted HR (95% CI)	*p* value	SGLT2 inhibitor (*n* = 11,670)	Non-SGLT2 inhibitor (*n* = 11,670)	Adjusted HR (95% CI)	*p* value
	81 (0.70)	1257 (0.75)	0.92 (0.73–1.44)	0.44	81 (0.70)	125 (1.07)	0.65 (0.45–0.89)	0.01

Data are presented as %. MACCE, major cardiac and cerebrovascular events; SGLT2, sodium glucose cotransporter 2; HR, hazard ratio; CI, confidence interval.

**Table 4:** Clinical outcomes for pneumonia and sepsis.

	Crude Population				Propensity Score-Matched Population			
Pneumonia	SGLT2 inhibitor (*n* = 12,401)	Non-SGLT2 inhibitor (*n* = 164,471)	Unadjusted HR (95% CI)	*p* value	SGLT2 inhibitor (*n* = 12,400)	Non-SGLT2 inhibitor (*n* = 12,400)	Adjusted HR (95% CI)	*p* value
	552 (4.45)	6915 (4.20)	1.06 (0.97–1.15)	0.20	552 (4.45)	544 (4.39)	1.03 (0.91–1.16)	0.62
Sepsis	SGLT2 inhibitor (*n* = 14,068)	Non-SGLT2 inhibitor (*n* = 188,432)	Unadjusted HR (95% CI)	*p* value	SGLT2 inhibitor (*n* = 14,063)	Non-SGLT2 inhibitor (*n* = 14,063)	Adjusted HR (95% CI)	*p* value
	33 (0.23)	575 (0.31)	0.76 (0.53–1.07)	0.13	33 (0.23)	39 (0.28)	0.78 (0.46–1.31)	0.36

Data are presented as %. SGLT2, sodium glucose cotransporter 2; HR, hazard ratio; CI, confidence interval.

## Data Availability

HIRA reserves the right to share data.

## Supplementary Materials

The following supporting information can be downloaded at: https://www.mdpi.com/article/10.3390/jcm13020431/s1, Figure S1. (A) propensity score, (B) covariate balance of the analysis of MACCE; Figure S2. Attrition of analysis about pneumonia; Figure S3. (A) propensity score, (B) covariate balance of the analysis of pneumonia; Figure S4. Kaplan-Meier curves for pneumonia in the (A) crude population and (B) propensity score matched population.

## Abbreviations

COVID	Coronavirus disease
SGLT2	Sodium glucose cotransporter 2
DM	Diabetes mellitus
HIRA	Health Insurance Review and Assessment Service of Korea
OMOP	Observational Medical Outcomes Partnership
CDM	Common data model
OHDSI	Observational Health Data Sciences and Informatics
MACCEs	Major adverse cardiac and cerebrovascular events

## References

[1] (2023). The Lancet. Long COVID: 3 Years in. Lancet.

[2] Gupta R., Hussain A., Misra A. (2020). Diabetes and COVID-19: Evidence, Current Status and Unanswered Research Questions. Eur. J. Clin. Nutr..

[3] Zheng Y.Y., Ma Y.T., Zhang J.Y., Xie X. (2020). COVID-19 and the Cardiovascular System. Nat. Rev. Cardiol..

[4] Fedorchenko Y., Zimba O. (2023). Comorbidities in the COVID-19 Pandemic: Scopus-Based Bibliometric Analysis. J. Korean Med. Sci..

[5] Raisi-Estabragh Z., Cooper J., Salih A., Raman B., Lee A.M., Neubauer S., Harvey N.C., Petersen S.E. (2022). Cardiovascular Disease and Mortality Sequelae of COVID-19 in the UK Biobank. Heart.

[6] Xie Y., Xu E., Bowe B., Al-Aly Z. (2022). Long-Term Cardiovascular Outcomes of COVID-19. Nat. Med..

[7] Braunwald E. (2022). SGLT2 Inhibitors: The Statins of the 21st Century. Eur. Heart J..

[8] Zelniker T.A., Wiviott S.D., Raz I., Im K., Goodrich E.L., Bonaca M.P., Mosenzon O., Kato E.T., Cahn A., Furtado R.H.M. (2019). SGLT2 Inhibitors for Primary and Secondary Prevention of Cardiovascular and Renal Outcomes in Type 2 Diabetes: A Systematic Review and Meta-Analysis of Cardiovascular Outcome Trials. Lancet.

[9] Sainsbury C., Wang J., Gokhale K., Acosta-Mena D., Dhalla S., Byne N., Chandan J.S., Anand A., Cooper J., Okoth K. (2021). Sodium-Glucose Co-Transporter-2 Inhibitors and Susceptibility to COVID-19: A Population-Based Retrospective Cohort Study. Diabetes Obes. Metab..

[10] Vitale R.J., Valtis Y.K., McDonnell M.E., Palermo N.E., Fisher N.D.L. (2021). Euglycemic Diabetic Ketoacidosis with COVID-19 Infection in Patients with Type 2 Diabetes Taking SGLT2 Inhibitors. AACE Clin. Case Rep..

[11] Kosiborod M.N., Esterline R., Furtado R.H.M., Oscarsson J., Gasparyan S.B., Koch G.G., Martinez F., Mukhtar O., Verma S., Chopra V. (2021). Dapagliflozin in Patients with Cardiometabolic Risk Factors Hospitalised with COVID-19 (DARE-19): A Randomised, Double-Blind, Placebo-Controlled, Phase 3 Trial. Lancet Diabetes Endocrinol..

[12] Kim J.W., Kim C., Kim K.H., Lee Y., Yu D.H., Yun J., Baek H., Park R.W., You S.C. (2023). Scalable Infrastructure Supporting Reproducible Nationwide Healthcare Data Analysis toward FAIR Stewardship. Sci. Data.

[13] Jiang G., Kiefer R.C., Sharma D.K., Prud’hommeaux E., Solbrig H.R. (2017). A Consensus-Based Approach for Harmonizing the OHDSI Common Data Model with HL7 FHIR. Stud. Health Technol. Inform..

[14] Fernández-de-las-Peñas C., Palacios-Ceña D., Gómez-Mayordomo V., Florencio L.L., Cuadrado M.L., Plaza-Manzano G., Navarro-Santana M. (2021). Prevalence of Post-COVID-19 Symptoms in Hospitalized and Non-Hospitalized COVID-19 Survivors: A Systematic Review and Meta-Analysis. Eur. J. Intern. Med..

[15] Elze M.C., Gregson J., Baber U., Williamson E., Sartori S., Mehran R., Nichols M., Stone G.W., Pocock S.J. (2017). Comparison of Propensity Score Methods and Covariate Adjustment: Evaluation in 4 Cardiovascular Studies. J. Am. Coll. Cardiol..

[16] Khunti K., Valabhji J., Misra S. (2023). Diabetes and the COVID-19 Pandemic. Diabetologia.

[17] Unsworth R., Wallace S., Oliver N.S., Yeung S., Kshirsagar A., Naidu H., Kwong R.M.W., Kumar P., Logan K.M. (2020). New-Onset Type 1 Diabetes in Children during COVID-19: Multicenter Regional Findings in the U.K. Diabetes Care.

[18] Kamrath C., Mönkemöller K., Biester T., Rohrer T.R., Warncke K., Hammersen J., Holl R.W. (2020). Ketoacidosis in Children and Adolescents with Newly Diagnosed Type 1 Diabetes during the COVID-19 Pandemic in Germany. JAMA.

[19] Maayah Z.H., Ferdaoussi M., Takahara S., Soni S., Dyck J.R.B. (2021). Empagliflozin Suppresses Inflammation and Protects against Acute Septic Renal Injury. Inflammopharmacology.

[20] Lee H.-F., Chan Y.-H., Chuang C., Li P.-R., Yeh Y.-H., Hsiao F.-C., Peng J.-R., See L.-C. (2023). Cardiovascular, Renal, and Lower Limb Outcomes in Patients with Type 2 Diabetes after Percutaneous Coronary Intervention and Treated with Sodium–Glucose Cotransporter 2 Inhibitors vs. Dipeptidyl Peptidase-4 Inhibitors. Eur. Heart J. Cardiovasc. Pharmacother..

[21] Tereshchenko L.G., Bishop A., Fisher-Campbell N., Levene J., Morris C.C., Patel H., Beeson E., Blank J.A., Bradner J.N., Coblens M. (2022). Risk of Cardiovascular Events after COVID-19. Am. J. Cardiol..

[22] Fox S.E., Li G., Akmatbekov A., Harbert J.L., Lameira F.S., Brown J.Q., Heide R.S.V. (2020). Unexpected Features of Cardiac Pathology in COVID-19 Infection. Circulation.

[23] McGonagle D., Plein S., O’Donnell J.S., Sharif K., Bridgewood C. (2020). Increased Cardiovascular Mortality in African Americans with COVID-19. Lancet Respir. Med..

[24] Cowie M.R., Fisher M. (2020). SGLT2 Inhibitors: Mechanisms of Cardiovascular Benefit beyond Glycaemic Control. Nat. Rev. Cardiol..

[25] Wu M.-Z., Chandramouli C., Wong P.-F., Chan Y.-H., Li H.-L., Yu S.-Y., Tse Y.-K., Ren Q.-W., Yu S.-Y., Tse H.-F. (2022). Risk of Sepsis and Pneumonia in Patients Initiated on SGLT2 Inhibitors and DPP-4 Inhibitors. Diabetes Metab..

[26] Bossi A.C., Forloni F., Colombelli P.L. (2020). Lack of Efficacy of SGLT2-i in Severe Pneumonia Related to Novel Coronavirus (NCoV) Infection: No Little Help from Our Friends. Diabetes Ther..

