# Supplementary material for: The Effect of Sodium–Glucose Cotransporter-2 Inhibitors on COVID-19 Patients with Type 2 Diabetes Mellitus: A Retrospective Cohort Study Using the Common Data Model
Source: J Clin Med. 2024 Jan 12;13(2):431. doi: 10.3390/jcm13020431 (PMC10815946; doi:10.3390/jcm13020431)
Supplement: Supplementary file 1 [file jcm-13-00431-s001.zip › jcm-2798396-supplementary.pdf]

Supplementary Materials

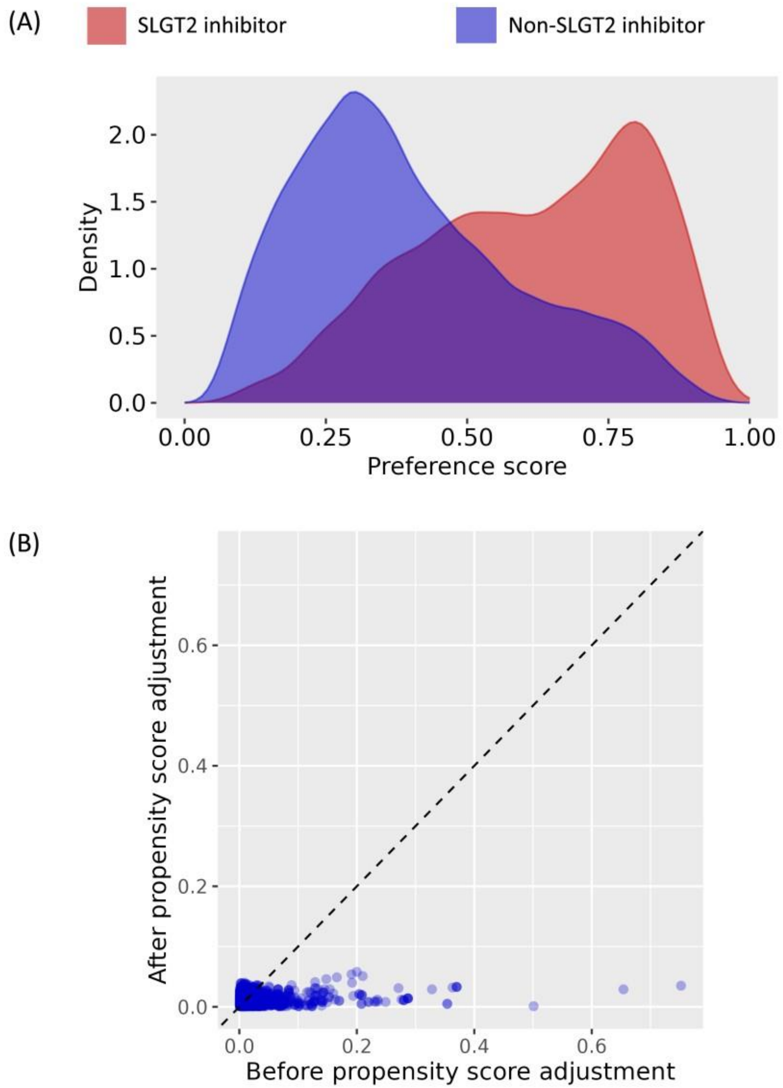

Figure S1. (A) propensity score, (B) covariate balance of the analysis of MACCE.

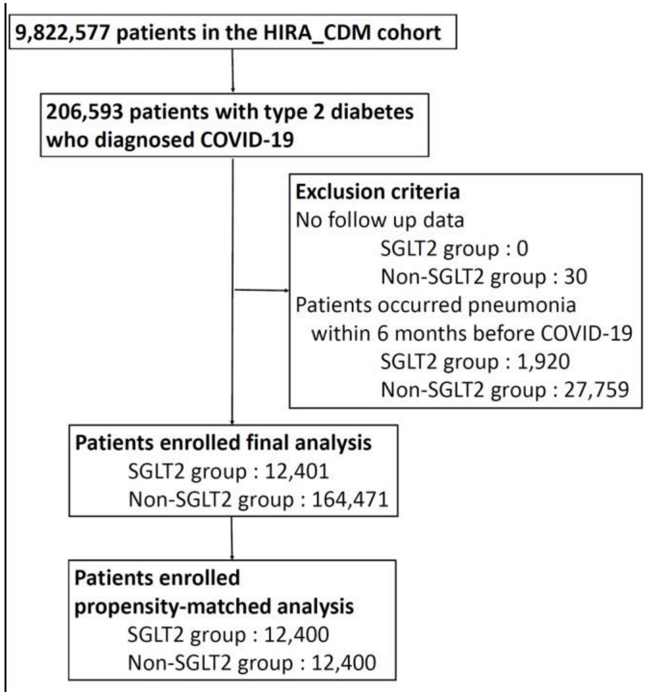

Figure S2. Attrition of analysis about pneumonia.

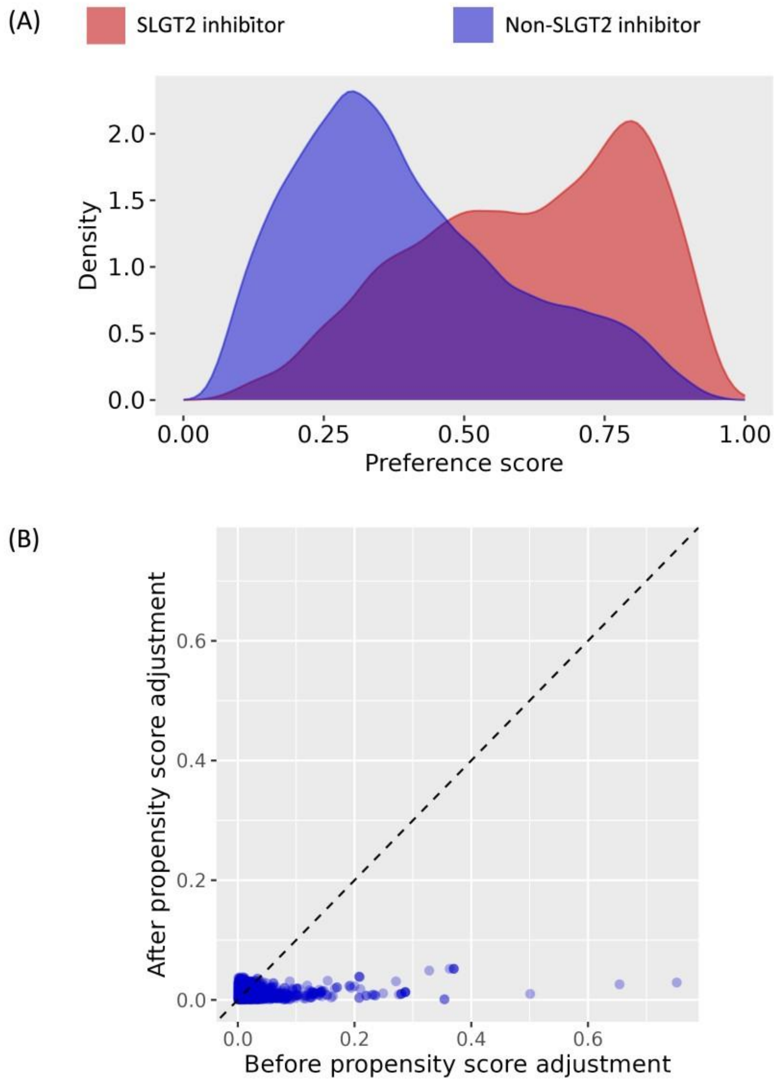

Figure S3. (A) propensity score, (B) covariate balance of the analysis of pneumonia.

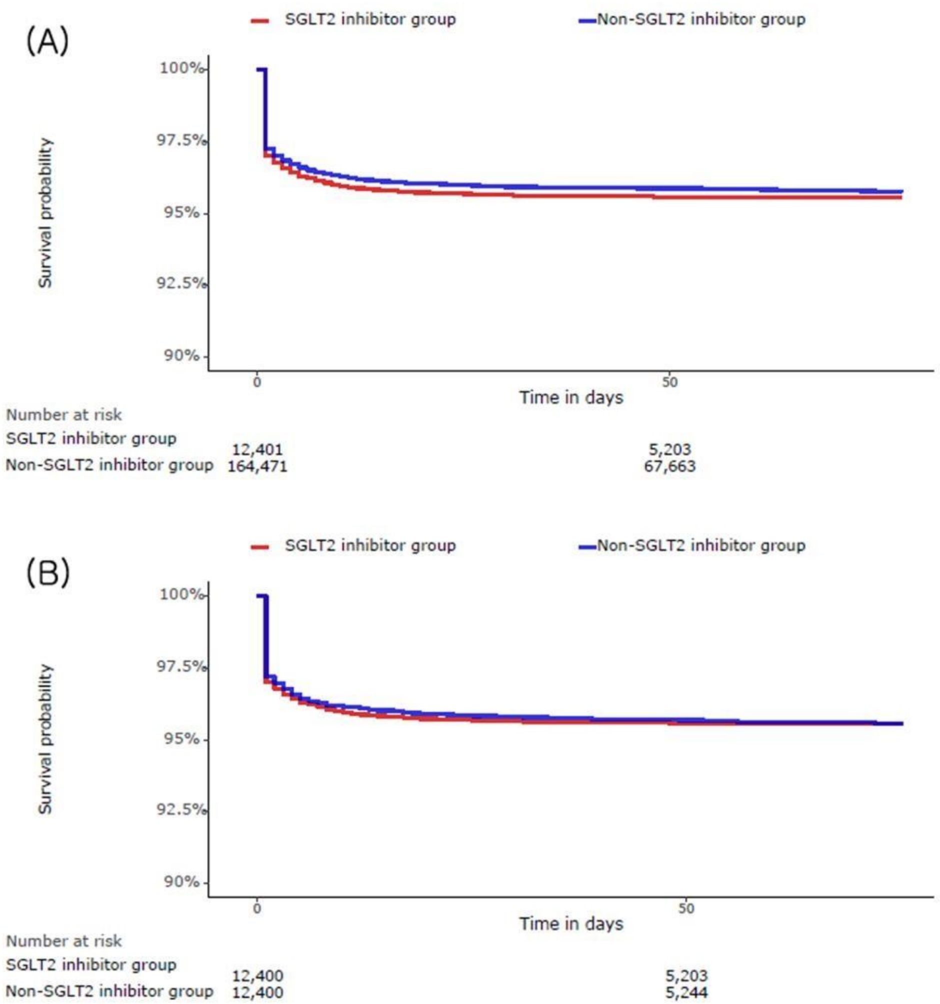

Figure S4. Kaplan-Meier curves for pneumonia in the (A) crude population and (B) propensity score matched population.
